# MicroRNA Expression Profile of Neural Progenitor-Like Cells Derived from Rat Bone Marrow Mesenchymal Stem Cells under the Influence of IGF-1, bFGF and EGF

**DOI:** 10.3390/ijms16059693

**Published:** 2015-04-29

**Authors:** Tee Jong Huat, Amir Ali Khan, Jafri Malin Abdullah, Fauziah Mohamad Idris, Hasnan Jaafar

**Affiliations:** 1 Department of Pathology, School of Medical Sciences, Universiti Sains Malaysia, Jalan Raja Perempuan Zainab II, 16150 Kubang Kerian, Kota Bharu, Kelantan, Malaysia; E-Mail: hasnan@usm.my; 2 Department of Neurosciences, School of Medical Sciences, Universiti Sains Malaysia, Jalan Raja Perempuan Zainab II, 16150 Kubang Kerian, Kota Bharu, Kelantan, Malaysia; E-Mails: amkhan@sharjah.ac.ae (A.A.K.); brainsciences@gmail.com (J.M.A.); 3 Department of Applied Biology, College of Sciences, University of Sharjah, Emirates of Sharjah, P.O. Box 27272, United Arab Emirates; 4 Sharjah Institute for Medical Research, University of Sharjah, Emirates of Sharjah, P.O. Box 27272, United Arab Emirates; 5 Director, Center for Neuroscience Services and Research, Universiti Sains Malaysia, Jalan Raja Perempuan Zainab II, 16150 Kubang Kerian, Kota Bharu, Kelantan, Malaysia; 6 Jabatan Neurosains, Hospital Universiti Sains Malaysia, Jalan Raja Perempuan Zainab II, 16150 Kubang Kerian, Kota Bharu, Kelantan, Malaysia; 7 Department of Medical Microbiology, School of Medical Sciences, Universiti Sains Malaysia, Jalan Raja Perempuan Zainab II, 16150 Kubang Kerian, Kota Bharu, Kelantan, Malaysia; E-Mail: fauziahmi@usm.my

**Keywords:** microRNA, IGF-1, microarray, bone marrow, mesenchymal stem cell, neural progenitor cell, proliferation, apoptosis

## Abstract

Insulin-like growth factor 1 (IGF-1) enhances cellular proliferation and reduces apoptosis during the early differentiation of bone marrow derived mesenchymal stem cells (BMSCs) into neural progenitor-like cells (NPCs) in the presence of epidermal growth factor (EGF) and basic fibroblast growth factor (bFGF). BMSCs were differentiated in three groups of growth factors: (A) EGF + bFGF, (B) EGF + bFGF + IGF-1, and (C) without growth factor. To unravel the molecular mechanisms of the NPCs derivation, microarray analysis using GeneChip^®^ miRNA arrays was performed. The profiles were compared among the groups. Annotated microRNA fingerprints (GSE60060) delineated 46 microRNAs temporally up-regulated or down-regulated compared to group C. The expressions of selected microRNAs were validated by real-time PCR. Among the 46 microRNAs, 30 were consistently expressed for minimum of two consecutive time intervals. In Group B, only miR-496 was up-regulated and 12 microRNAs, including the let-7 family, miR-1224, miR-125a-3p, miR-214, miR-22, miR-320, miR-708, and miR-93, were down-regulated. Bioinformatics analysis reveals that some of these microRNAs (miR-22, miR-214, miR-125a-3p, miR-320 and let-7 family) are associated with reduction of apoptosis. Here, we summarize the roles of key microRNAs associated with IGF-1 in the differentiation of BMSCs into NPCs. These findings may provide clues to further our understanding of the mechanisms and roles of microRNAs as key regulators of BMSC-derived NPC maintenance.

## 1. Introduction

Bone marrow derived mesenchymal stem cells (BMSCs) are mesodermal multipotent stem cells with self-renewal capacities and possess the ability to differentiate beyond their lineage into endoderm and even ectoderm cells *in vitro* [1,2]. Our previous study showed that BMSCs could be differentiated into neural progenitor-like cells (NPCs) under a specifically induced microenvironment [3]. We found a combination of epidermal growth factor (EGF), basic fibroblast growth factor (bFGF), and insulin-like growth factor 1 (IGF-1) could significantly improve the quality of the derived NPCs, as the addition of IGF-1 enhances cell proliferation and survivability compared to the published protocol used only EGF and bFGF. An identical combination of growth factors was also reported to provide an optimal niche for embryonic striatal stem cell maintenance [4]. However, the molecular mechanism of IGF-1 addition on BMSC-derived NPCs maintenance is still unclear. We believed that alteration to gene expression by microRNAs play important role in the enhancement of cellular activities.

MicroRNAs are short noncoding RNA with 18 to 22 nucleotides that regulates gene expression at posttranscriptional levels by base pairing with targeted messenger RNA (mRNA) [5]. MicroRNAs bind on the 3'-untranslated region of mRNA by perfect base pairing, leading to mRNA cleavage. In contrast, binding with imperfect base pairing may cause translational repression or deadenylation [5]. A single microRNA may regulate hundreds of target mRNAs and single target mRNA may be regulated by several microRNAs. Therefore, microRNA-mRNA interaction forms a complex gene regulatory network. MicroRNAs also regulate genes at the transcriptional level by modulating DNA methylation and histone modification. For instance, miR-10 is required for hypermethylation in gastric cancer, and the mechanism was predicted by targeting the *HOXA1* gene [6]. Similarly, miR-874, a putative tumor suppressor in human cancers, can target histone deacetylase 1 in head and neck squamous cell carcinoma and contributes to cell proliferation [7]. Taken together, microRNAs can be considered important players in the control of epigenetics modification.

MicroRNAs have also emerged as powerful regulators of diverse biological processes, including cell differentiation [8,9], proliferation [10] and apoptosis [11,12]. MicroRNAs are also involved as key modulators of neuronal development, neuroplasticity, and disease pathogenesis, such as neurodegenerative disease and traumatic brain injuries [11,13]. The substantial value of microRNAs has been reported for medical diagnostic and prognostic determination, which eventually will lead to novel therapeutic intervention [14]. In addition, microRNAs have critical roles in stem cell differentiation and the derivation of induced pluripotent stem cells [15].

To elucidate the functions of microRNAs in stem cell differentiation, global profiling, such as microRNA microarray, microRNA sequencing, real-time PCR, and next generation sequencing of microRNA [16], may be carried out to find differentially and uniquely expressed microRNAs involved in the differentiation of BMSCs into neural lineages. Several microRNA expression studies have been carried out, such as the elucidation of genes involved between the mouse frontal cortex and hippocampus [17] and microRNA expression pattern changes in spinal-cord injury [18]. However, no study to date has characterized microRNA expression patterns in BMSC-derived NPCs under the influence of IGF-1. This information is critical since changes in cellular physiology, such as apoptosis and growth rate, are closely related to their microRNA-mRNA interactome within cells.

Microarray of microRNA can reveal differential expression of several microRNAs and microRNA family. We hypothesized that microRNA family may act as a whole in regulating specific cellular functions and pathways during the differentiation of BMSCs into NPCs. Therefore, the aim of this study was to identify the biological functions and pathways that might be involved due to the expression of key microRNAs during the differentiation of BMSCs into NPCs under the effect of IGF-1.

## 2. Results

### 2.1. Effect of Insulin-Like Growth Factor 1 (IGF-1) in the Enhancement of Bone Marrow Derived Mesenchymal Stem Cells (BMSCs)-Derived Neural Progenitor-Like Cells (NPCs)

**Figure 1:**
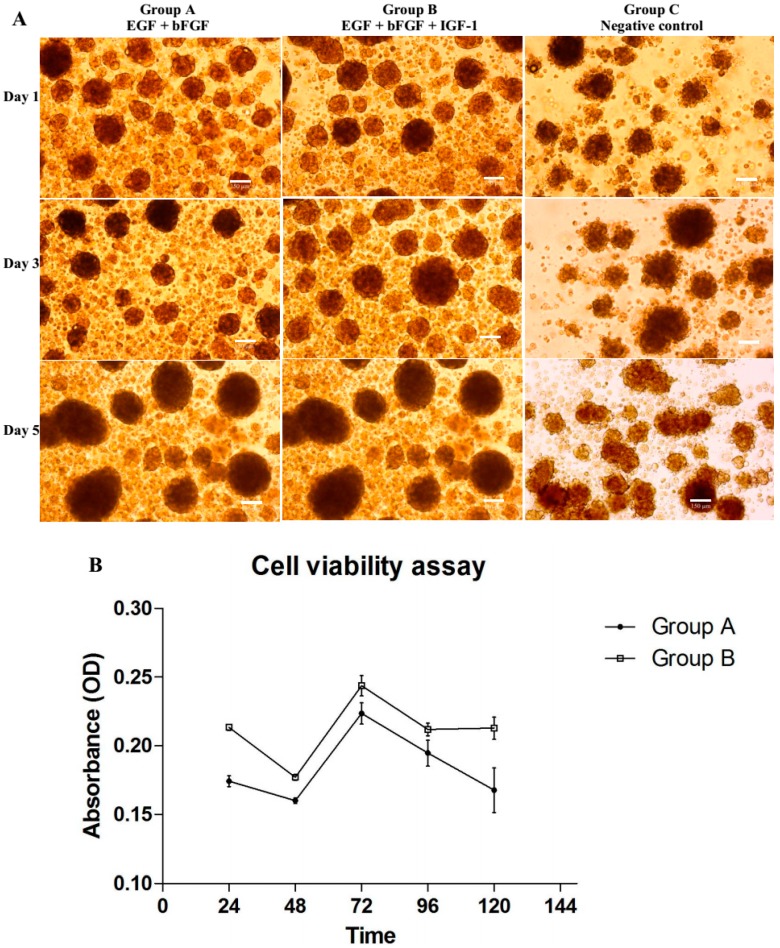
(**A**) Differentiation of bone marrow derived mesenchymal stem cells (BMSCs) into neural progenitor-like cells (NPCs) at day 1, 3 and 5 showing differing in sizes and morphology of neurospheres under different combination of growth factors. Cells were cultured in NeuroCult^®^ NS-A neural basal media under serum free condition. Group A (EGF + bFGF), a published protocol of combination of growth factors required for neuronal differentiation of BMSCs; Group B (EGF + bFGF + IGF-1), an enhanced protocol of neuronal differentiation; and Group C (neural basal media without growth factor,) served as negative control of the experiment. Neurospheres with irregular shape was observed only in group C. Images were viewed under inverted light microscope with 100× magnification, Scale bar = 150 µm; (**B**) Cells proliferation were studied at five time intervals (24, 48, 72, 96 and 120 h) with 4 h incubation each with CellTiter 96^®^ Aqueous One solution reagent (*n* = 6). Group B contained significant higher proportions of vial cells (*p* = 0.0098 with 95% CI) compared to Group A. The data represented as optical density (OD) at 540 nm in mean ± standard deviation.

**Figure 2:**
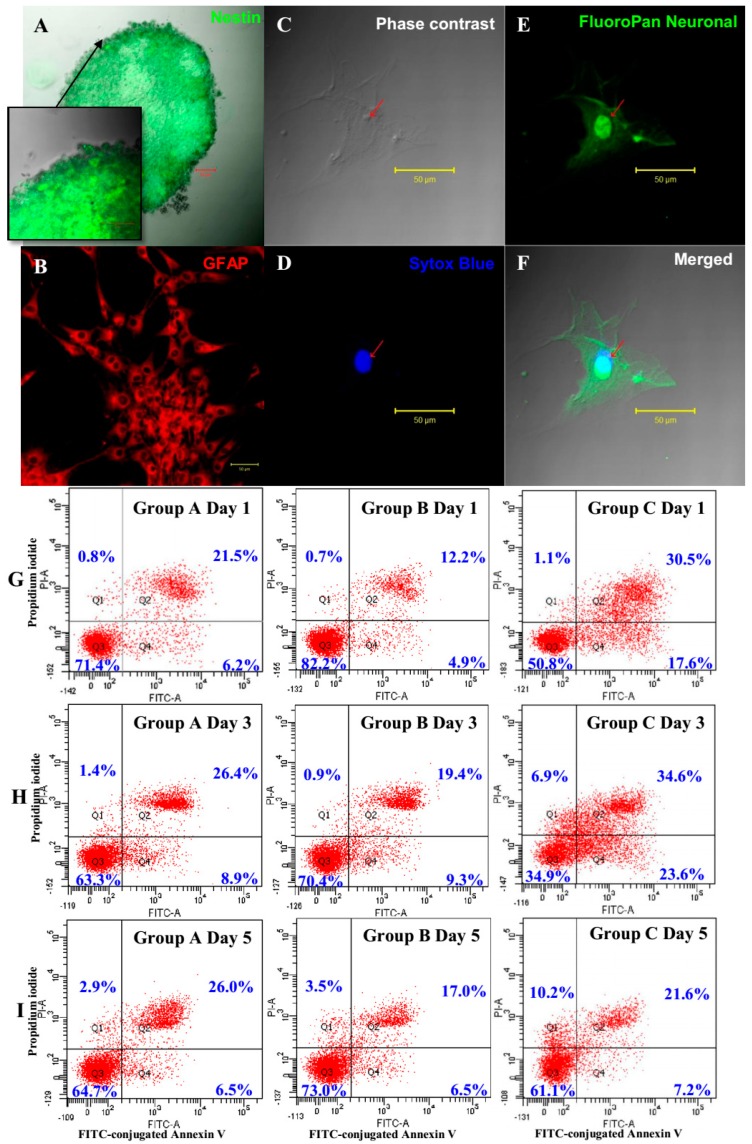
(**A**–**F**) Immunocytochemical staining of terminally differentiated BMSC-derived NPCs from Group B (EGF + bFGF + IGF-1) into neurons and astrocytes. (**A**) Neurosphere shows positive expression against nestin antibody; (**B**) Astrocytes were stained with glial fibrillary acidic protein (GFAP) primary antibody and counterstained with Cy3 secondary antibody; (**C**) Phase contrast image of differentiated cells; (**D**) Nucleus was stained with Sytox Blue; (**E**) Neurofilament body was stained against FluoroPan neuronal marker conjugated with fluorescein isothiocyanate (FITC); (**F**) Overlay of images (**C**–**E**). All images are viewed at 40× magnification under a confocal microscope; Scale bars = 50 µm; (**G**–**I**) Flow cytometry analysis of apoptosis assay comparing between treatment groups (Group A and B) and negative control (Group C). (**G**) Dots plot showing the apoptotic effect on Day 1 of differentiation; (**H**) Day 3 and (**I**) Day 5. Plot in first quadrant, Q1—Annexin^−^/PI^+^ necrotic cells; Q2—Annexin^+^/PI^+^ late apoptotic cells; Q3—Annexin^−^/PI^−^ live cells and Q4—Annexin^+^/PI^−^ early apoptotic cells.

BMSCs from group A and B under the influence of their respective growth factors combination formed free-floating neurosphere-like bodies after 24 h of treatment (Figure 1A). The combinatorial effects of the growth factors (EGF + bFGF + IGF-1) could be observed clearly in the enhancement of cell viability (Figure 1B). The proliferation rate of NPCs under IGF-1 treatment was significantly higher than that of the Group A (*p* < 0.01) with 95% confidence interval. Neurospheres under IGF-1 treatment showed largest diameters among the groups and generally oval in shape as compared to negative control (Figure 1A). Floating bodies in Group C showed irregular shape (Figure 1A) and partly remained as BMSCs or undergone differentiation [3]. Dark cores at the center of the neurospheres were observed at Day 5, indicating that the neurospheres had reached the maximum size (200–250 µm). NPCs generated by Group B strongly expressed nestin (Figure 2A) and can be terminally differentiated into cells with glial (Figure 2B) and neural (Figure 2F) phenotypes. Cell viability data was supported by the apoptotic and cell death activity of the cells detected using Annexin V and propidium iodide. The IGF-1-treated NPCs showed better survivability (Figure 2G), as compared to the Group A, by maintaining a higher number of live cells and lower apoptotic (Figure 2H) and necrotic activities (Figure 2I). To sum up, addition of IGF-1 could significantly improve the production of healthier and functional NPCs as compared to the published protocol of using only the combination of EGF and bFGF.

**Figure 3:**
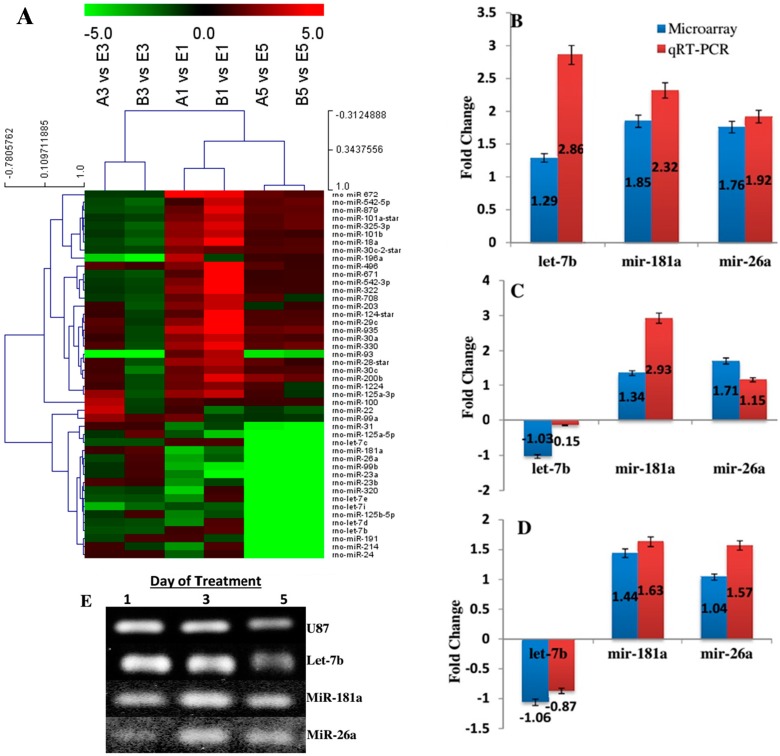
(**A**) Hierarchal clustering of differentially expressed microRNAs. Forty-six differentially expressed microRNAs (for up- and down-regulation, respectively) compared to the negative control group in at least one sample were clustered hierarchically. Each rows represent individual microRNAs. The expression ratio representing colour ranges from green (low) to red (high), as indicated by the scale bar. The column bar chart shows the relative expression level of selected microRNAs by qRT-PCR (right column, white) as compared to the fold changes of the corresponding microRNA (left column, grey); (**B**) MicroRNAs expression of Group B *versus* Group A on the first day of treatment; (**C**) Group B *versus* Group A on the third day of treatment; (**D**) MicroRNAs expression of on the fifth day of treatment. U87 was used as housekeeping gene. Bars represent the mean ± standard error of three independent experiments; and (**E**) qRT-PCR product was confirmed by gel electrophoresis at 90 V for 40 min.

### 2.2. Identification of MicroRNAs by Affymetrix MicroRNA Array

To compare microRNA expression patterns among the different treatments, we analyzed the microRNA expression of Group A (EGF and bFGF), Group B (EGF, bFGF, and IGF-1), and Group C (without growth factor) with Affymetrix GeneChip microRNA 2.0 arrays. From each group, three biological replicates were analyzed on independent arrays. Among the 389 mature microRNAs present on the array chip, 46 microRNAs with consistent expression were detected in all groups over the experimental period (Day 1, Day 3 and Day 5) (Figure S1). Figure 3A shows the detected microRNAs displaying the highest variance (top 30th percentile) throughout the differentiation (expression baseline adjusted to the mean of control sample, Group C), and the 46 differentially expressed microRNAs are listed in Table S1.

In accordance with the clustering profile of microRNAs, two patterns were observed (up- and down-patterns). MicroRNAs with consistent expression (up-regulated or down-regulated) at least on two consecutive treatment periods were taken into account (Table 1 and Table 2). MicroRNAs with inconsistent differential expression are listed in Table S2. In the group treated with EGF and bFGF, 8 microRNAs were down-regulated and 14 microRNAs were consistently up-regulated compared to Group C throughout the three time intervals of treatment. However, BMSC-derived NPCs with addition of IGF-1 showed 12 microRNAs which include miR-22, miR-1224, miR-125a-3p, miR-214, miR-320, miR-708 and miR-93 were consistently down-regulated and only miR-496 remained up-regulated compared to Group C from Day 1 to Day 5. The let-7 family (let-7b, let-7c, let-7d, let-7e and let-7i) were constantly down-regulated in both groups.

**Table 1:** List of microRNAs up-regulated and down-regulated due to EGF + bFGF. Down-regulation of microRNAs in two consecutive treatment days (Day 3 and 5) was considered as down-regulated and *vice versa*.

Down-Pattern				Up-Pattern			
miRNAs	Treatment Day			miRNAs	Treatment Day		
	1	3	5		1	3	5
**miR-23a**	−2.52	−1.03	−27.66	**miR-100**	1.32	3.01	1.11
**miR-26a**	−3.16	−1.39	−9.71	**miR-1224**	1.77	1.13	1.33
**miR-320**	−4.04	−1.39	−39.65	miR-124*	1.46	1.02	1.33
**let-7d**	−2.67	−1.35	−78.22	**miR-125a-3p**	2.48	2.54	1.19
**let-7e**	-2.71	−1.62	−115.16	**miR-200b**	1.84	1.26	2.34
**let-7i**	−1.50	−3.55	−25.15	miR-28*	2.61	1.21	1.61
**miR-99b**	−3.47	−1.02	−19.24	**miR-29c**	2.26	1.49	1.33
**miR-125a-5p**	−1.63	−1.02	−26.80	**miR-30a**	2.22	1.02	1.40
**miR-125b-5p**	−2.57	−1.09	−42.83	**miR-30c**	1.72	1.16	1.26
**let-7b**	1.12	−1.54	−193.29	**miR-330**	2.64	1.38	1.56
**let-7c**	1.27	−1.56	−121.62	**miR-496**	2.16	1.49	1.47
**miR-191**	1.18	−1.31	−17.43	**miR-935**	3.31	1.35	1.75
**miR-93**	1.65	−5.56	−4.53				

**Table 2:** List of microRNAs up-regulated and down-regulated due to EGF + bFGF + IGF-1. Down-regulation of microRNAs in two consecutive treatment days (Day 3 and 5) was considered as down-regulated and *vice versa*.

Down-Pattern				Up-Pattern			
miRNAs	Treatment Day			miRNAs	Treatment Day		
	1	3	5		1	3	5
**miR-22**	−2.20	−1.22	−1.72	**miR-496**	4.53	1.14	1.06
**let-7i**	−1.79	−2.06	−22.39				
**let-7b**	1.44	−1.59	−204.17				
**let-7c**	1.33	−1.55	−218.82				
**let-7d**	1.37	−1.50	−75.57				
**let-7e**	1.21	−1.55	−91.88				
**miR-1224**	2.26	−1.46	−1.06				
**miR-125a-3p**	3.32	−1.66	−1.06				
**miR-214**	1.34	−1.19	−37.19				
**miR-320**	1.04	−1.21	−28.70				
**miR-708**	3.30	−1.55	−1.02				
**miR-93**	2.95	−14.42	−4.10				

**Table 3:** List of microRNAs comparing between Group B (EGF + bFGF + IGF-1) and A (EGF + bFGF).

Down-Pattern				Up-Pattern			
miRNAs	Treatment Day			miRNAs	Treatment Day		
	1	3	5		1	3	5
**let-7b**	1.29	−1.03	−1.06	**let-7e**	3.27	1.05	1.25
**miR-100**	−1.32	−3.97	−1.01	**miR-125b-5p**	1.68	1.31	1.01
**miR-196a**	−4.23	−3.76	−1.10	**miR-181a**	1.85	1.34	1.44
**miR-22**	−2.51	−4.16	−1.60	**miR-24**	1.89	1.03	3.07
**miR-99a**	−2.25	−1.76	−1.09	**miR-26a**	1.76	1.71	1.04
****				**miR-320**	4.22	1.15	1.38

In addition, several microRNAs in Groups A and B that showed up-regulation on Day 1 but then their expression went down consistently at Day 3 and 5 of treatment. These were also listed and considered in the analysis. Interestingly, miR-22, miR-1224 and miR-125-3p were initially up-regulated under EGF and bFGF treatment, but reversed their quantitative expression in the presence of IGF-1. In addition, miR-214 and miR-708 were expressed inconsistently in Group A while their expression went down in Group B.

### 2.3. Quantitative RT-PCR for miRNA Validation

To validate the microRNA microarray expression data, a qRT-PCR assay was conducted to confirm the expression levels of three randomly selected microRNAs (let 7-b, miR-181a, and miR26a). The qRT-PCR analysis confirmed that let-7b, miR-181a, and miR-26a were significantly up-regulated on Day 1 after differentiation (Figure 3B). The expression of let-7b was down-regulated on both Day 3 (Figure 3C) and Day 5 (Figure 3D) after induction whereas miR-181a and miR-26a remained up-regulated. Real-time expression was confirmed by gel electrophoresis analysis and the gel bands were photographed (Figure 3E).

### 2.4. Target Prediction Analysis

To identify microRNA targeted mRNAs, target prediction for all microRNAs consistently up-regulated or down-regulated in both groups (Table 1 and Table 2) were performed using the validated target search engine in the miRWalk database [19]. The list of predicted genes are listed in Table S3. A total of 2518 genes were targeted by 29 consistently expressed microRNAs, including two star microRNAs (miR-28-star and miR-124-star). The number of target genes predicted for each microRNA varied from 2 (miR-330) to 436 (let-7c) (Figure S2). The majority of the targeted genes were specific for the regulation of cell proliferation (18.9%), programmed cell death (18.6%), and apoptosis (18.1%) (Data not shown).

### 2.5. GO Analysis of MicroRNA Targets

**Figure 4:**
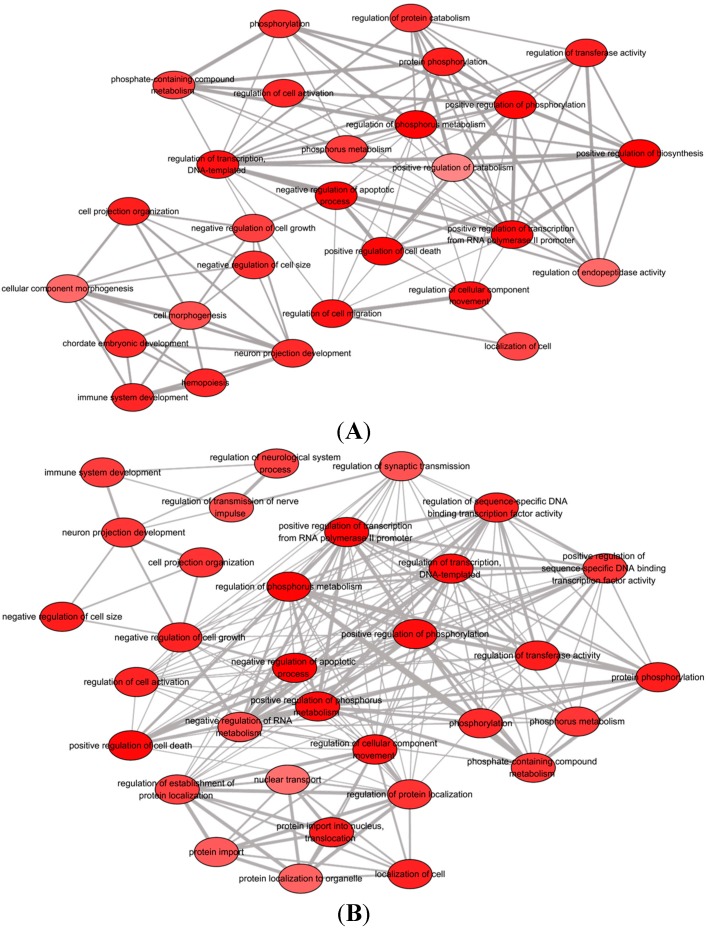
A network composed of statistically significant (FDR < 0.01) non-redundant GO terms associated with down-regulation of microRNAs in (**A**) Group A, EGF + bFGF and (**B**) Group B, EGF + bFGF + IGF-1. Figures were constructed by REVIGO software. The color density of each GO term is proportional to statistical significance.

To understand the biological functions of the two patterns of microRNAs, a simple GO analysis was performed using DAVID software (National Cancer Institute, Frederick, MD, USA). Up- and down-pattern gene sets from both treatment groups, including the gene set specific for the let-7 family, were considered in the analysis. Only the gene set for down-regulated microRNAs from both groups showed statistically significant GO terms (*p*-value < 0.05, FDR < 0.01), whereas no significant GO term for up-regulated microRNAs was seen in the respective species (*Rattus norvegicus*). The top 10 statistically significant GO terms (FDR < 0.01) with κ similarity threshold set at 0.85 (highest stringency = 1.00) are shown in Table 4 (for full list of enriched GO terms, see Table S4). The GO terms were listed based on their respective enrichment score in descending order. Each enrichment score was calculated based on the geometric mean in log scale of the *p*-value (Fisher exact/ EASE score) for the members of a corresponding annotation cluster. The enriched GO terms were then entered into the REViGO software (Rudjer Boskovic Institute, Zagreb, Croatia) to remove redundant terms and generate functional relationship network structure. Figure 4A and 4B show the biological process networks altered by the down-regulation of microRNAs in Group A (EGF + bFGF) and Group B (EGF + bFGF + IGF-1), respectively.

**Table 4:** Top 10 GO terms associated with down-regulation of microRNAs in groups A and B.

Group	GO Terms	Biological Processes	* *p*-Value	** FDR
**A(EGF + bFGF)**	GO: 0043067	Regulation of programmed cell death	3.81 × 10^−19^	6.71 × 10^−16^
	GO: 0010941	Regulation of cell death	4.45 × 10^−19^	7.98 × 10^−16^
	GO: 0042981	Regulation of apoptosis	8.72 × 10^−18^	1.53 × 10^−14^
	GO: 0009891	Positive regulation of biosynthetic process	1.27 × 10^−18^	2.23 × 10^−15^
	GO: 0031328	Positive regulation of cellular biosynthetic process	3.96 × 10^−18^	6.97 × 10^−15^
**B(EGF + bFGF + IGF-1)**	GO: 0043067	Regulation of programmed cell death	3.42 × 10^−19^	6.10 × 10^−16^
	GO: 0010941	Regulation of cell death	4.12 × 10^−19^	7.34 × 10^−16^
	GO: 0042981	Regulation of apoptosis	6.27 × 10^−18^	1.12 × 10^−14^
	GO: 0043066	Negative regulation of apoptosis	1.25 × 10^−16^	2.00 × 10^−13^
	GO: 0043069	Negative regulation of programmed cell death	1.94 × 10^−16^	4.00 × 10^−13^
	GO: 0060548	Negative regulation of cell death	2.12 × 10^−16^	4.00 × 10^−13^

*p-value was calculated using Fisher’s exact test. ** FDR corrections were based on Benjamini-Hochberg procedure in DAVID program.

### 2.6. GeneMANIA Association Network and GO: 0043066 Analyses

**Figure 5:**
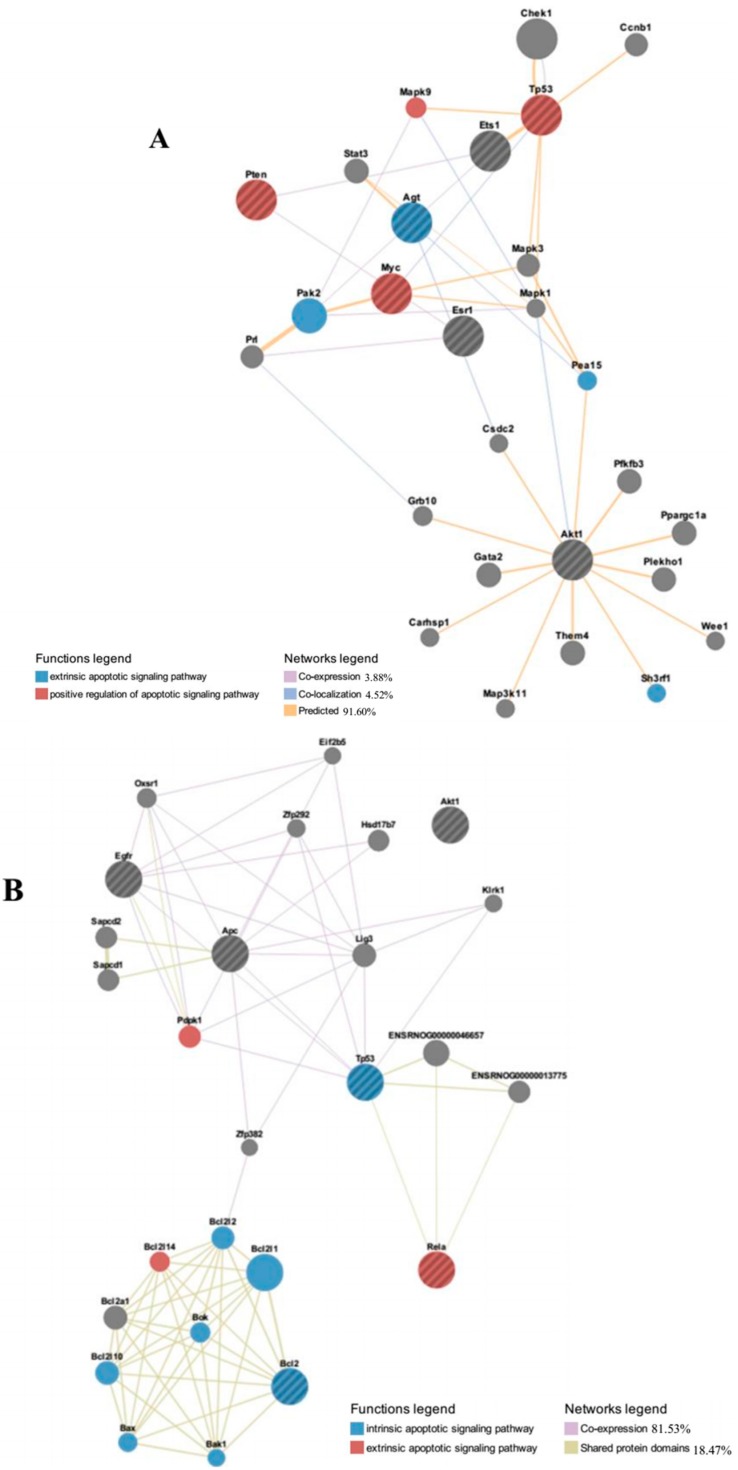

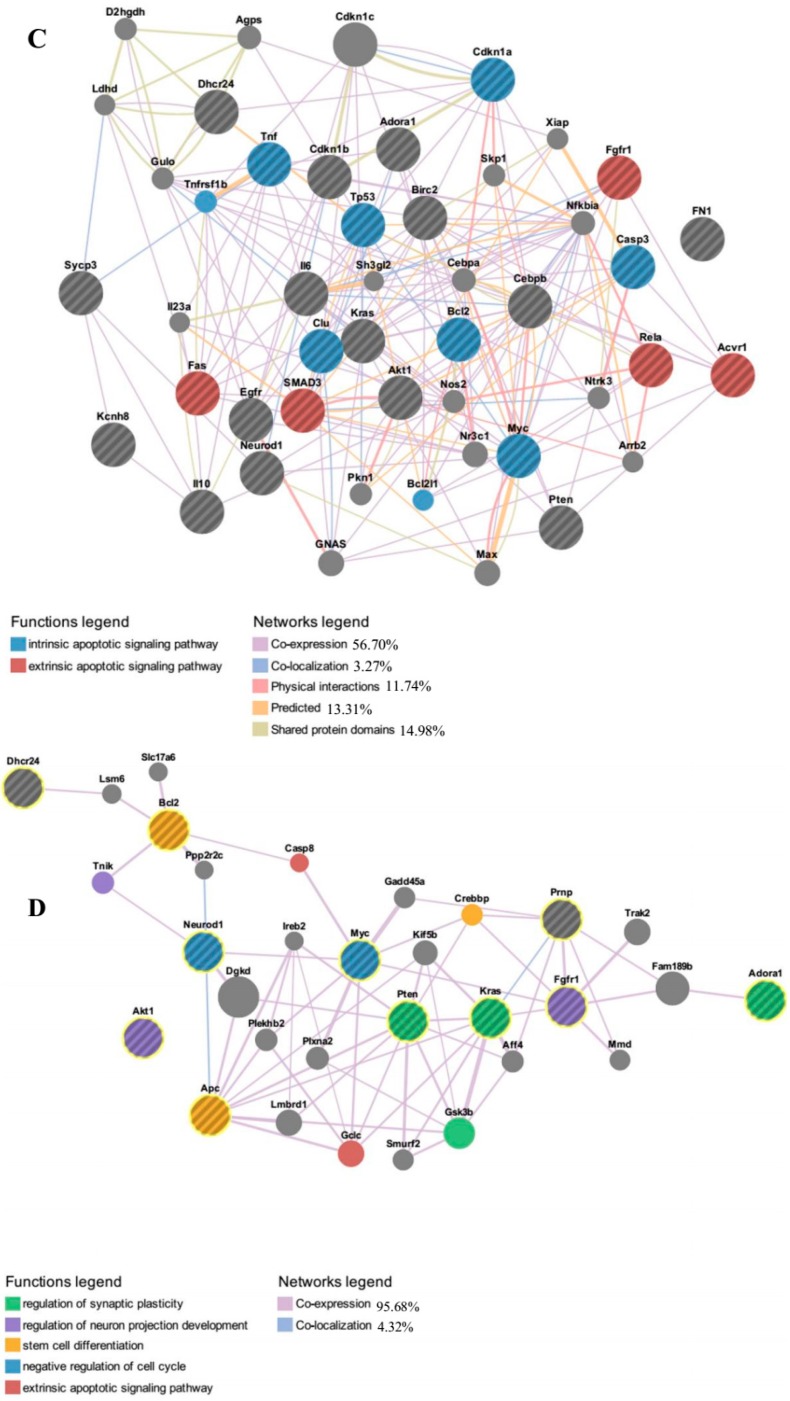

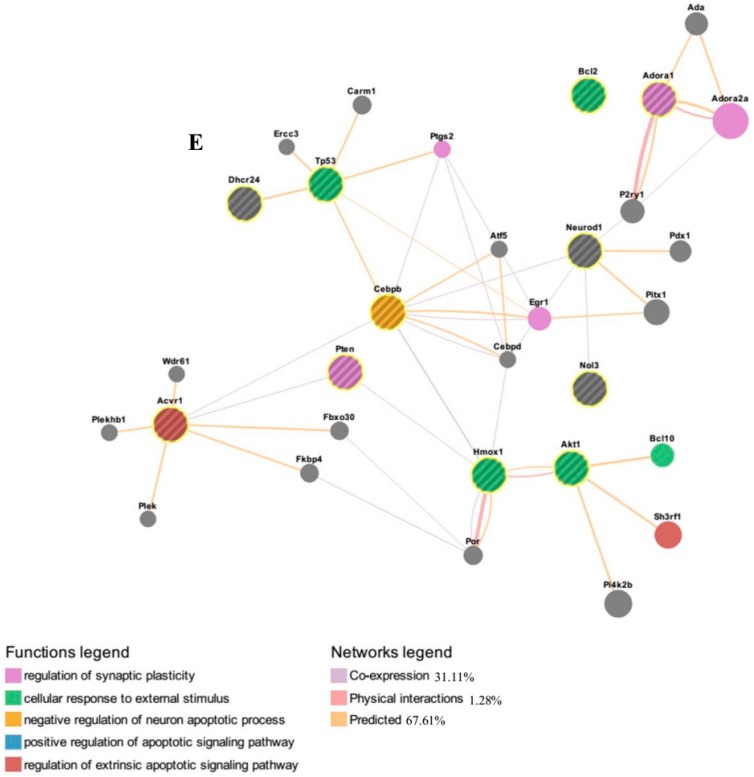
Identification of molecular functional networks and gene ontology analysis of rat’s network genes. The genes up-regulated by down-regulation of miR-22 (**A**); miR-125a-3p (**B**); let-7 family (**C**); miR-214 (**D**); and miR-320 (**E**) were analyzed using GeneMANIA web tool with default weighting method (*i.e.*, weighting based to maximize connectivity between input genes). The edges are indicated based on similar gene expression across conditions (co-expression), interact in a protein-protein interaction (physical interaction), similar protein domain (shared protein domains), expression in the same tissue or gene product identified in the same cellular location, and prediction of functional relationships between genes (predicted).

In order to predict the potential interactions between key differentially regulated microRNAs and targeted mRNAs involved in the reduction of apoptotic activity in Group B and with additional genes outside of the searched network, we plotted an extended rat network associated with GO term 0043066—negative regulation of apoptosis (Figure 5A–E). MiR-1224, miR-708 and miR-93 were excluded from analysis since there is no mRNA associated with negative regulation of apoptosis. We used GeneMANIA web tool that searches a very large set of publicly available functional network data, which include proteomic and genomic interactions, pathways, co-expression and co-localization of genes and protein domain similarity [20]. Query genes for individual microRNA are listed in Table 5. The major targeted genes by all or four out of five key microRNAs (miR-22, miR-214, miR-125a-3p, miR-320 and let-7 family) included Akt1, Tp53, Pten and Bcl2. Information about search parameters, statistics used and additional outputs are shown in Table S5.

**Table 5:** List of predicted target genes involved in negative regulation of apoptosis.

MicroRNAs	Query Genes
**miR-22**	*Myc*; *Ets1*; *Tp53*; *Agt*; *Esr1*; *Pten*; *Akt1*
**miR-214**	*Bcl2*; *Adora1*; *Myc*; *Neurod1*; *Dhcr24*; *Kras*; *Fgfr1*; *Apc*; *pcgfr1*; *Prnp*; *Akt1*
**miR-125a-3p**	*Bcl2*; *Egfr*; *Tp53*; *Apc*; *Akt1*; *Rela*
**miR-320**	*Bcl2*; *Adora1*; *Acvr1*; *Neurod1*; *Dhcr24*; *Tp53*; *Hmox1*; *Nol3*; *Pten*; *Akt1*; *Cebpb*
**Let-7 Family**	*Cdkn1a*; *Tnf*; *Bcl2*; *Adora1*; *Egfr*; *Myc*; *Il10*; *Acvr1*; *Sycp3*; *Neurod1*; *Dhcr24*; *Cdkn1b*; *SMAD3*; *Kras*; *ras3*; *Neurod1Birc2*; *Tp53*; *Kcnh8*; *FN1*; *Fgfr1*; *Clu*; *Fas*; *Pten*; *Akt1*; *Rela*; *Cebpb*

### 2.7. Pathway Analysis of Target mRNA Focusing on Cell Proliferation and Survivability

We assessed the predicted target genes of the down-regulated microRNAs with the KEGG database. The significantly overrepresented pathways with an FDR of less than 0.01 are shown in Table S6. Additionally, the statistical significant pathways (FDR < 0.01) involving cell proliferation and survivability related to this study are listed in Table 6. The significance levels of the related pathways were compared with pathways targeted by specific let-7 microRNAs independently to exclude the possibility that those pathways are due to let-7 microRNAs alone. The majority of the pathways were related to the enhancement of cell proliferation and prevention of apoptosis.

**Table 6:** Pathways of predicted target genes for down-regulated microRNAs in Group A, Group B and Let-7 Family. *n* = Number of targeted genes in a specific pathway. Statistical significant was expressed in term of false discovery rate (FDR).

KEGG ID	Pathways	Group A		Group B		Let-7 Family	
		*n*	* FDR	*n*	* FDR	*n*	* FDR
**rno05200**	Pathways in cancer	40	4.19 × 10^−18^	39	3.50 × 10^−15^	29	5.40 × 10^−12^
**rno04010**	MAPK signaling pathway	24	3.01 × 10^−6^	26	1.20 × 10^−6^	18	3.84 × 10^−4^
**rno04210**	Apoptosis	13	1.37 × 10^−4^	12	3.72 × 10^−3^	10	5.13 × 10^−3^
**rno04012**	ErbB signaling pathway	10	n.s.	12	3.72 × 10^−3^	10	5.14 × 10^−3^
**rno04350**	TGF-β signaling pathway	14	1.68 × 10^−5^	12	4.18 × 10^−3^	11	6.03 × 10^−4^
**rno04620**	Toll-like receptor signaling pathway	13	2.62 × 10^−4^	12	6.57 × 10^−3^	9	n.s.
**rno04060**	Cytokine-cytokine receptor interaction	19	8.69 × 10^−5^	17	1.13 × 10^−2^	13	n.s.
**rno04660**	T cell receptor signaling pathway	13	n.s.	12	n.s.	11	5.46 × 10^−3^

* FDR corrections were calculated using the Benjamini-Hochberg procedure; n.s.: Statistically no significant.

## 3. Discussion

The present profiling work was the first to decipher the microRNA expression patterns in adult rat BMSC-derived NPCs. Microarray-based microRNA profiling is a common method to discover the differentially and uniquely expressed microRNAs. This procedure is the first step in the screening process of overexpressed microRNAs that in turn regulate gene expression.

Both bFGF and EGF have been shown to be crucial factors in differentiating BMSCs into neural lineage cells [21]. IGF-1 is another polypeptide important for nervous system development [22]. By stimulating the IGF-1 receptor, IGF-1 acts as an important mitogenic factor to promote neural cell proliferation and survival *in vitro* and *in vivo* [23].

In our previous study, we confirmed that a combination of bFGF, EGF and IGF-1 could induce the neuronal differentiation (NPCs) of BMSCs and significantly (*p* < 0.05) enhance BMSCs-derived NPCs viability in undifferentiated state [3]. However, the molecular mechanisms such as microRNAs profiling, associated with this enhanced differentiation remain to be elucidated. Therefore, in this study, we described the microRNA signatures of NPCs (P1) specifically at Day 1, 3 and 5 after induction. These signatures showed the pattern of microRNA changes during differentiation. However, one of the main limitations in deciphering the mechanism of epigenetic modification and maintenance of NPCs is the complexity of biological pathways associated with regulating microRNAs. Therefore, it is difficult to reveal the specific biological pathways based on the analysis of a handful of predicted genes.

Among the 389 rat-specific microRNA probes on the GeneChip^®^ microRNA 2.0 array, we discovered 46 microRNAs up-regulated or down-regulated due to the effects of growth factors. However, microRNAs with inconsistent expression throughout the treatment days from Groups A and B were excluded from analysis. Only microRNAs differentially expressed at least on two consecutive treatment days were taken into account.

There are 30 microRNAs (Table 1 and Table 2) consistently expressed in two patterns (up-pattern or down-pattern) from Day 1 to Day 5. Both up- and down-patterned microRNAs were observed in Group A; however, in the presence of IGF-1, the majority of microRNAs were down-regulated and only miR-496 was up-regulated. Since the down-regulation of microRNAs may cause the up-regulation of targeted genes, we hypothesized that the presence of IGF-1 triggers the expression of certain genes by down-regulating key microRNAs (miR-1224, miR-125a-3p, miR-214, miR-22, miR-320, miR-708, and miR-93), which in turn enhance NPCs proliferation and survivability.

In Groups A and B, let-7 family microRNAs were observed to be consistently down-regulated. These data suggest contribution of different members of one microRNA cluster or family act as a whole in regulating specific signaling pathways. MicroRNAs from miR-8 family and miR-34 family were reported to be involved in the regulation of ceramide signaling pathway in the frontal cortex and dopamine signaling pathway in the hippocampus respectively [17]. In addition, Itesako *et al.* concluded that down-regulation of miR-195/497 cluster contributed to bladder cancer progression by targeting *BIRC5* and *WNT7A* genes [24]. Therefore, our finding is supported by previous experiments regarding signaling pathway targeted by either microRNA families or clusters. Besides, Cimadamore *et al.* reported that LIN28 binds to precursor let-7 microRNA and blocks the production of mature let-7i microRNAs, inhibiting neuronal differentiation by targeting *MASH1* and *NGN1* genes [25]. This process suggests that the down-regulation of the let-7 microRNA family promotes cell proliferation during early neurogenesis.

miR-93, a microRNA frequently associated with TGF-β signaling in controlling cell cycle arrest [26], cell proliferation, and differentiation [27], was also down-regulated in both Groups A and B. Moreover, we discovered that miR-1224 and miR-125a-3p, which were initially up-regulated in Group A, became down-regulated at day 3 and 5 in Group B post-induced with IGF-1. Both miR-1224 and miR-125a-3p play important roles in maintaining cell proliferation and survivability. The down-regulation of miR-1224 positively regulates TNF-α gene expression [28], which strongly influences NPC survival, proliferation, and neuronal differentiation [29,30].

The down-regulation of miR-125a-3p has been associated with a reduction in apoptosis by targeting p53 mRNA and an increase in cell proliferation and migration by up-regulating Fyn expression [31,32]. Stronger p53 protein expression has been associated with the induction of apoptosis and inhibition of cell proliferation [33]. Similarly, the reduction of Fyn and its downstream proteins causes cell cycle arrest at G2/M stage, consequently, reducing cell viability and migration [32]. These data support our findings that the down-regulation of mir-125-3p reduced apoptosis and increased cell proliferation possibly by a p53- and Fyn-regulated manner.

Furthermore, the introduction of IGF-1 triggers the down-regulation of several microRNAs (miR-214, miR-22, miR-320 and miR-708) with their inconsistent differential expression in Group A. The inhibition of miR-214 has been reported to decrease the level of apoptosis in HeLa cells [34]. Yu *et al.* reported that miR-22 is a pivotal candidate for gene therapy in cerebral ischemic injury due to its neuroprotective effect [35]. The down-regulation of miR-708 was reported to be involved in the enhancement of cell proliferation [36] and the expression of miR-708 has been reported to be associated with PI3K/Akt signaling pathways [37]. In summary, a group of microRNAs with individual roles forms a complex functional network, and together they contribute to the enhancement of BMSC-derived NPCs.

Differences in functional involvement of down-regulated microRNAs in Groups A and B were measured using GO and pathway analyses. Biological relevant of each GO terms were determined by enrichment score. Top-ranked annotation groups indicated by higher enrichment score are indicated as more significant biologically [38,39]. Both gene groups in A and B were generally associated with cell growth and survival signaling functions (Table 4 and Figure 4). The GO term “regulation of programmed cell death” (GO: 0043067) was overrepresented in both groups, resulting from the activation of endogenous cellular processes that play important roles in cell epigenetic modification [40]. The regulation of non-apoptotic programmed cell death is most likely due to the effects of combinatorial EGF and bFGF in which the synergistic treatment of both growth factors modify the BMSCs fate into a neural lineage.

Zhao *et al.* reported that EGF and bFGF trigger the expression of miR-9, which targets the nuclear receptor TLX controlling NSC proliferation and fate determination [41]. Chuang and Jones reported that small interfering RNAs closely related to microRNAs are involved in the histone modification and DNA methylation that regulate the mechanism of epigenetic modification [42]. However, the gene list of Group B (EGF + bFGF + IGF-1) showed a higher important level with an enrichment score of 15.76 (34 genes involved) than did Group A (EGF + bFGF) with an enrichment score of 13.31 (29 genes involved) in GO term “negative regulation of apoptotic process” (GO: 0043066). These data indicate that the down-regulation of key microRNAs in Group B due to IGF-1 triggers some yet to be determined biological processes that reduce apoptotic process. Our GO analysis data is consistent with our apoptosis assay findings described earlier.

Furthermore, functional networks analysis of up-regulated genes associated with GO term “negative regulation of apoptosis process” by the algorithm GeneMANIA. It revealed several genes such as *Akt1* (v-Akt murine thymoma viral oncogene homolog 1), *Tp53* (tumor protein p53), *Pten* (phosphatase and tensin homolog) and *Bcl2* (B-cell lymphoma 2). Protein kinase B or Akt, a key protein involved in the activation of PI3K-Akt pathway and is crucial in promoting cell survivability [43], is inhibited by the key microRNAs (miR-22, miR-214, miR-125a-3p, miR-320 and let-7 family) that are down-regulated with the addition of IGF-1. Chen *et al.* reported that down-regulation of miR-133b significantly overexpressed *Akt1* mRNA, which increased T24 bladder cancer cell proliferation and reduced cell apoptosis [44]. Overexpression of *Akt1* gene also triggers the expression of *Pten*, an antagonist of the PI3K pathway [45]. It has been reported that up-regulation of miR-93 stimulated cell proliferation and inhibited apoptosis through Akt pathway by targeting *Pten* and *Cdkn1a* [46]. Wang *et al.* showed experimental evidence of miR-21 inhibition triggered the overexpression of *Pten* and *Ptpn14*, which was a result of suppressed intrahepatic cholangiocarcinoma cell proliferation and growth [47].

Moreover, down-regulation of key microRNAs also triggers the expression of anti-apoptotic gene *Bcl-2* and tumor suppressor *p53*. Down-regulation of miR-451 and miR-885-5p in neuron-like cells has been shown to increase *Bcl-2* expression and lead to reduction of apoptosis activity [48]. Similarly, Lin *et al.* showed that inhibition of miR-34a restored the down-regulated expression of anti-apoptotic gene *Bcl-2* and thus decreased apoptotic rate in pancreatic β-cell [49]. In contrast, *Tp53* has been described as essential mediator of cell cycle arrest or pro-apoptotic gene, which has strong genetic and biochemical ties with *Bcl-2* [50]. Yoo *et al.* reported that inhibition of cell growth may occur through up-regulation of p53 which leading to G1/S cell cycle arrest [51]. Zhang *et al.* also reported that SOX4 transcription factor inhibits Glioblastoma cell growth partly via the activation of p53-p21 signaling which induces G0/G1 cell cycle arrest [52]. Therefore, taking all together we could postulate that down-regulation of key microRNAs due to IGF-1 induction simultaneously activate the agonist and antagonist of cell growth, which on the one hand, enhanced cell proliferation, but on the other hand, might control the rate of cell proliferation.

Cell growth and survival-related pathways, including the pathways in cancer (KEGG 5200), MAPK signaling (KEGG 4010), apoptosis (KEGG 4210), cytokine-cytokine receptor interaction (KEGG 4060), and TGF-β (KEGG 4350) cell cycle, were significantly (*p* < 0.05, FDR < 0.01) enriched in the predicted target genes of down-regulated microRNAs in Groups A and B. This suggests that the down-regulation of microRNAs due to growth factors is biologically functional (Table 5). Up-regulated genes due to the down-regulation of microRNAs by EGF, bFGF, and IGF-1 treatment were predicted to target EGFR, FGFR, and IGFR, respectively, via cytokine-cytokine receptor interaction in the cancer pathway. This leads to the activation of Ras protein, which is involved in MAPK and PI3K-Akt signaling pathways. The MAPK [53,54] and PI3K-Akt pathways [43] have been widely reported to be involved in cell proliferation and evading apoptosis. Moreover, the effects of growth factors on the Ras/MAPK/ERK signaling pathway inhibit the TGF-β pathway, which blocks cells from apoptosis and neurogenesis. At the same time, the activated MAPK pathway also triggers the expression of c-Myc regulator genes, which inhibit cyclin-dependent kinase inhibitor 2B on the TGF-β pathway, and leads to cell cycle arrest at G1 phase. In addition, the involvement of the apoptosis pathway showed that down-regulated microRNAs regulate the effect of IAP and Bcl-2, which suppress apoptotic activity [55,56]. Therefore, we postulate that the crosstalk between the MAPK and TGF-β pathways is the underlying mechanism of maintaining NPCs in undifferentiated state and producing less apoptotic cells [57].

The other functionally related pathways identified were the ErbB (KEGG 4010) and Toll-like signaling pathways (KEGG 4620). EGF targets the ErbB-1 (EGFR) receptor in the ErbB signaling pathway, which similarly leads to the activation of the MAPK and PI3K-Akt signaling pathways and, thus, regulates cell proliferation. The ErbB signaling pathway has been shown to promote the proliferation of neoplastic Schwann cells [58]. The Toll-like signaling pathway was reported to modulate the NPC cell-fate decision and provide neuroprotective effects through the activation of Toll-like receptors (TLRs), such as TLR2 and TLR4 [59,60]. In summary, each pathway is interconnected with each other and, as a result, promotes NPCs proliferation and reduces cells apoptosis.

## 4. Materials and Methods

### 4.1. Animals

Male Sprague-Dawley rats of age 4 to 6 weeks were obtained from the Animal Research and Service Centre of Universiti Sains Malaysia (USM). All rats had access to water and food pellets *ad libitum*. The animal ethics clearance for all experimental procedures involving animals was approved by the board of the Animal Ethics Committee of USM (USM/Animal Ethics Approval/(59) (196)).

### 4.2. Primary Cells Isolation and Culture

Bone marrow tissues were isolated from the rats’ tibia and femoral bones according to previously described procedures [3]. Briefly, animals were euthanized by intraperitoneal injection of ketamine (100 mg/mL) and xylazine (100 mg/mL) (both from Ilium Troy Laboratory, Blacktown, Australia). The central canal of the bone was flushed with 2 mL of Dulbecco Modified Eagle’s Media (DMEM) supplemented with 10% fetal bovine serum (FBS) and 1% penicillin-streptomycin (pen/strep) (all from Gibco, Life Technologies, Carlsbad, CA, USA) to extrude the marrow tissue. Mononuclear cells were purified using Ficoll-Paque PREMIUM solution (GE Healthcare Bioscience, Uppsala, Sweden) and the extracted cells were cultured on T25 cm^2^ flasks in a 37 °C humidified chamber with 5% CO_2_. After 24 h, the non-adherent cells were removed and the attached cells were allowed to grow. Complete media for BMSC proliferation (DMEM + 20% FBS + 1% pen/strep + 1% nonessential amino acids) was used to maintain the cells. The cells were subcultured until passage 3 (P3) and were then characterized by Fluorescein isothicyanate (FITC)-conjugated CD90 mouse monoclonal antibody (1:200; Thermo Scientific, Waltham, MA, USA), a surface marker for BMSCs. Translineage differentiation was conducted using fourth generation (P4) of cells.

### 4.3. Translineage Differentiation of BMSCs into NPCs

BMSCs at P4 were differentiated into NPCs as previously described [3]. Briefly, BMSCs were dissociated and induced into neurogenesis with NeuroCult^®^ NS-A proliferation media (STEMCELLS Technologies, Vancouver, BC, Canada) supplemented with three combinations of growth factors: Group A (10 ng/mL EGF + 10 ng/mL bFGF); Group B (10 ng/mL EGF + 10 ng/mL bFGF + 10 ng/mL IGF-1); and Group C, negative control involving no growth factors. Epidermal growth factor (EGF, catalog # GF155), fibroblast growth factor basic (bFGF, catalog # GF003) and insulin-like growth factor (IGF-1, catalog # GF121) were purchased from Merck Millipore, Billerica, MA, USA. Cells were maintained for 1 week in the incubator at 37 °C with 5% CO_2_. Fresh growth factors were added by changing half the media on the second and fourth days of differentiation.

### 4.4. NPC Proliferation Assay

Viability of the NPCs from each group was determined by colorimetric method using CellTiter 96^®^ Aqueous One solution (Promega, Fitchburg, WI, USA). Briefly, BMSCs were counted using a Countess^®^ automated cell counter (Life Technologies, Carlsbad, CA, USA). Then they were seeded into 96-well cell culture plates at 1 × 10^3^ cells per well within their respective differentiation media. The cells were incubated with CellTiter 96^®^ Aqueous One solution at 37 °C for 4 h prior to optical density measurement. Spectrophotometric measurements were obtained from each group of cells at 12, 24, 48, 72 and 96 h according to the exact procedure suggested in the instruction manual.

### 4.5. NPC Survivability Assay

The survivability of NPCs under the influence of growth factors was assessed using FITC-conjugated Annexin V and propidium iodide stains (both from BD Pharmingen, San Diego, CA, USA) according to the manufacturer’s protocol. Apoptotic activities among groups were compared by quantitative data acquired from flow cytometry analysis.

### 4.6. MicroRNA Isolation and Quality Control

Total RNA from nine cell populations, including Group A (Day 1, Day 3, Day 5), Group B (Day 1, Day 3, Day 5), and Group C (control) (Day 1, Day 3, Day 5), were isolated for microRNA analyses. Total RNA from each population was isolated in triplicate from three independent experiments with an miRNeasy mini kit (Qiagen, Hilden, Germany) as per the manufacturer’s instruction. The quantity of RNA was assessed using a NanoDrop™ 2000 Spectrophotometer (Thermo Scientific, Driesch, Germany). The quality of the total RNA and small RNA was checked using a 2200 Agilent TapeStation System (Agilent Technologies, Santa Clara, CA, USA). The system detected 28S and 18S ribosomal RNA ratios and generated an RNA integrity number equivalent (RIN^e^) value. The existence of a small RNA population was confirmed from the gel snapshot (Figure S3). Only samples with a 28S/18S more than 1.2, RIN^e^ value more than 9.0, and detectable small RNA strands were used for microarray analysis (Table S7). Total RNA samples were aliquoted and stored at −80 °C until used.

### 4.7. Hybridization and Microarray Scanning

A total of 27 (3 groups × 3 days × 3 biological replicates) RNA samples (1000 ng) from the three groups of cells were polyadenylated and ligated to a biotin signal molecule with a FlashTag™ biotin HSA RNA labeling kit (Genisphere, Hatfield, PA, USA). A colorimetric Enzyme Linked Oligosorbent Assay (ELOSA) was run simultaneously to confirm successful biotin labeling. The biotinylated RNA samples were then hybridized to Affymetrix GeneChip^®^ microRNA 2.0 arrays (Affymetrix, Santa Clara, CA, USA) for 16 hours protected from light at 48 °C with a rotation speed of 60 rpm. After hybridization, the arrays were washed and stained according to the FS450_0003 fluidics protocol, and then they were scanned using an Affymetrix 3000 7G scanner.

The probe cell intensity files (*.CEL files) generated by Affymetrix GeneChip^®^ Command Console^®^ software were imported into microRNA QC Tool software, version 1.1.1, to determine data quality. Expression Console™ software was used to process the *.CEL files into probe level summarization files (*.CHP files) using a robust multi-array (RMA) detection algorithm workflow. The *.CHP files were further analyzed using Transcriptome Analysis Console (TAC) software, version 2.0, to identify and visualize the differentially expressed genes. All software products were downloaded from Affymetrix homepage (http://www.affymetrix.com/).

### 4.8. MicroRNA Microarray Data Analysis

The microRNA microarray data analysis was performed with the GeneSpring GX 12.6 software (Agilent Technologies, Santa Clara, CA, USA). The raw *.CEL files were imported into the software prior to the analysis. The default RMA detection above background (RMA-DABG) setting was used to background correct, normalize, and summarize all expression values for each gene on each array. This dataset is available through the Gene Expression Omnibus (GEO) database (http://www.ncbi.nlm.nih.gov/) with the accession number GSE60060. A two-way Analysis of variance (ANOVA) was then used to calculate the p-value for each microRNA probe comparing among the groups in three different time points. The significance threshold value to define the up-regulation and down-regulation of microRNAs was at fold-change more than or equal to 1.5 (at least one out of three treatment days) with an equivalent p-value of less than 0.05. From these microRNAs, we analyzed the microRNA expression patterns obtained by three interpretation approaches. First, Group A versus Group C analyzed the microRNA expression pattern due to the effects of EGF and bFGF, as the combination of these growth factors are essential for neural lineage differentiation. Second, Group B versus Group C analyzed the microRNA expression pattern due to EGF, bFGF, and IGF-1. Third, Group B versus Group A analyzed the fold changes between the two groups due to IGF-1 addition. Hierarchical clustering analysis of differentially expressed microRNAs was performed using MultiExperiment Viewer (MeV), version 4.0, software (Dana-Farber Cancer Institute, Boston, MA, USA).

### 4.9. MicroRNA-qPCR Assay

MicroRNA expressions were validated using quantitative reverse transcription polymerase chain reaction (qRT-PCR) according to the manufacturer’s protocol (all from Applied Biosystems, Life Technologies Co.; Waltham, MA, USA). Total RNA samples were transcribed to cDNA with a TaqMan^®^ MicroRNA Reverse Transcription kit. The TaqMan^®^ Universal PCR Master Mix II with no Uracil-N glycoslyase was used and qRT-PCR was performed using the following thermal protocol with the TaqMan^®^ Real-Time PCR system: 95 °C for 10 min, 40 cycles of 95 °C for 15 s, and 60 °C for 60 s. Three microRNAs were selected for validation based on the comparison between Groups A and B (Table 3). Of these, let-7b was from the down-regulated list, and miR-26a and miR-181a were from the up-regulated list. Forward and reverse primers for mature microRNA miR-26a-3p (Assay ID: 462650_mat; miR-seq: 5'-CCUAUUCUUGGUUACUUGCAC-3'), miR-181a-3p (Assay ID: 000516; miR-seq: 5'-ACCAUCGACCGUUGAUUGUACC-3'), and let-7b-3p (Assay ID: Rn03465224_pri; miR-seq: 5'-CUAUACAACCUACUGCCUUCCC-3') were used for qRT-PCR amplification. Small nucleolar RNA U87 (Assay ID: 001712; 72 bp) was selected as an endogenous control gene. The relative expression of microRNA was calculated using Relative Expression Software Tool (REST) 2009, V2.0.13, (http://rest.gene-quantification.info) [61]. The mean difference between the cycle thresholds value (*C*_t_) of each microRNA and the U87 endogenous control within each sample group was calculated (−∆*C*_t_). The fold change in microRNA expression was determined by calculating the difference between the mean ∆*C*_t_ of the Group B and Group A samples (∆∆*C*_t_), and the results were expressed as fold-change (2^−∆∆*C*t^) [18]. The independent two-tailed *t*-test was used to compare the *C*_t_ value changes among groups, and *p*-value of less than 0.05 was considered statistically significant.

### 4.10. Target Prediction

Target mRNAs was predicted by the miRWalk [19] database (http://www.ma.uni-heidelberg.de/apps/zmf/mirwalk/), which takes into account the improvement of prediction accuracy by multiple computational methods. A validated target module on miRWalk database was used to predict the genes targeted by identified key microRNAs. This module hosts all experimentally verified microRNAs information associated with their genes, pathways, and also the information on proteins known to be involved in microRNA processing. List of targeted mRNAs in the form of official gene symbol were extracted from the miRWalk prediction results for further analysis.

### 4.11. Gene Ontology (GO) Analysis

Predicted gene lists for both up- and down-patterns of microRNA clusters were uploaded to the Database for Annotation, Visualization, and Integration Discovery (DAVID) software, version 6.7, (http://david.abcc.ncifcrf.gov) for simple GO analysis as previously described [39,62]. Up-regulated or down-regulated microRNAs with consistent expression at least on two consecutive treatment days were considered in the analysis. The *p*-value for each GO-term was calculated using Fisher’s exact test. The Benjamini-Hochberg procedure was applied for increased stringency. The resultant statistical significant GO-terms were entered into the Reduce + Visualize Gene Ontology (REViGO) software (Rudjer Boskovic Institute, Zagreb, Croatia) to construct a meaningful network structure by excluding redundant subsets of GO terms. Functional similarity among GO terms was measured based on the SimRel score [63].

### 4.12. MicroRNA-mRNA Functional Network Analysis

GeneMANIA web tool (http://genemania.org/) [64] was used to construct the molecular functional networks of the genes targeted by specific microRNAs. A default weighting method (maximized genes connectivity strategy) was used for calculations. All down-regulated microRNAs in Group B (IGF-1 treated) were considered in the analysis. Briefly, predicted target genes of individual microRNA were clustered using DAVID software with high stringency (κ similarity threshold = 0.85). Genes related to GO: 0043066 negative regulation of apoptosis process were analyzed using GeneMANIA web tool and functional network images were developed. The database version for these searches was 1 June 2014 and application version 3.1.2.8.

### 4.13. Pathway Analysis

A pathway prediction analysis was performed using DAVID [62]. Similar to GO analysis, significant pathways were identified based on the input list of predicted genes and corrected *p*-value. The gene lists from Group A, Group B, and the let-7 family were considered in the analysis. Only highly significant pathways with a *p*-value less than 0.05 and FDR less than 0.01 were listed as potential pathways for further analysis. The pathway information used in this study was generated from the Kyoto Encyclopedia of Genes and Genomes (KEGG, http://www.genome.jp/kegg/) online database.

### 4.14. Statistical Analysis

The two-way ANOVA was done for the microRNA profiles with the GeneSpring software. The Student’s *t*-test was performed to compare the gene expression value between two groups and all data were expressed as the mean ± SD. A *p*-value of less than 0.05 was considered statistically significant in all experiments. However, the *p*-values for GO and pathway analyses were calculated based on Fisher’s exact test and corrections according to the Benjamini-Hochberg procedure were applied.

## 5. Conclusions

In conclusion, the results of the present study indicate that the introduction of IGF-1 in the basic neural induction media with EGF and bFGF enhanced the quality of BMSC-derived NPCs in terms of cell proliferation and survivability by the down-regulation of key microRNAs. These fundamental findings may provide clues to further our understanding of the mechanisms and roles of microRNAs as key regulators of BMSC-derived NPC maintenance and their prospect in future medical application, especially in neural regenerative medicine.

## Supplementary Materials

Supplementary materials can be found at http://www.mdpi.com/1422-0067/16/05/9693/s1.
