# Supplementary material for: MicroRNA Expression Profile of Neural Progenitor-Like Cells Derived from Rat Bone Marrow Mesenchymal Stem Cells under the Influence of IGF-1, bFGF and EGF
Source: Int J Mol Sci. 2015 Apr 29;16(5):9693–718. doi: 10.3390/ijms16059693 (PMC4463612; doi:10.3390/ijms16059693)
Supplement: Supplementary file 1 [file ijms-16-09693-s001.zip › ijms-75630-Supplementary Information/ijms-75630-Supplementary Information.pdf]

Supplementary Information

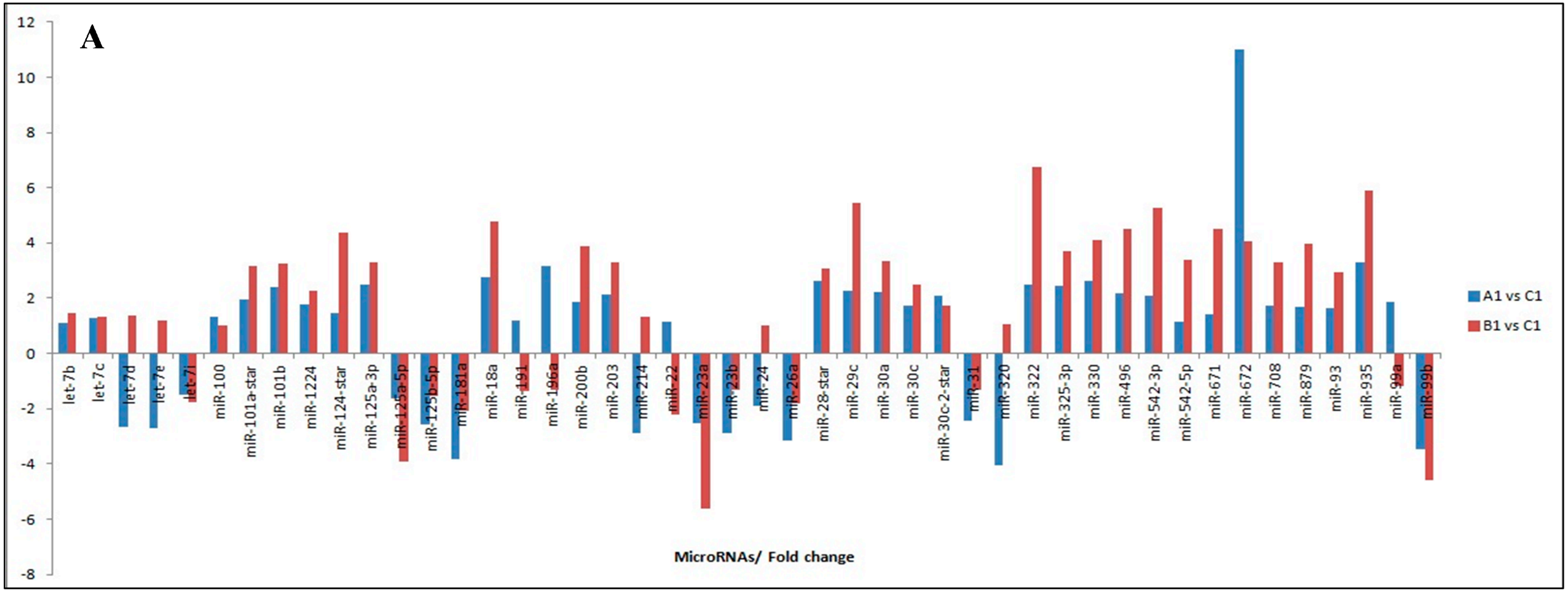

Figure S1. *Cont.*

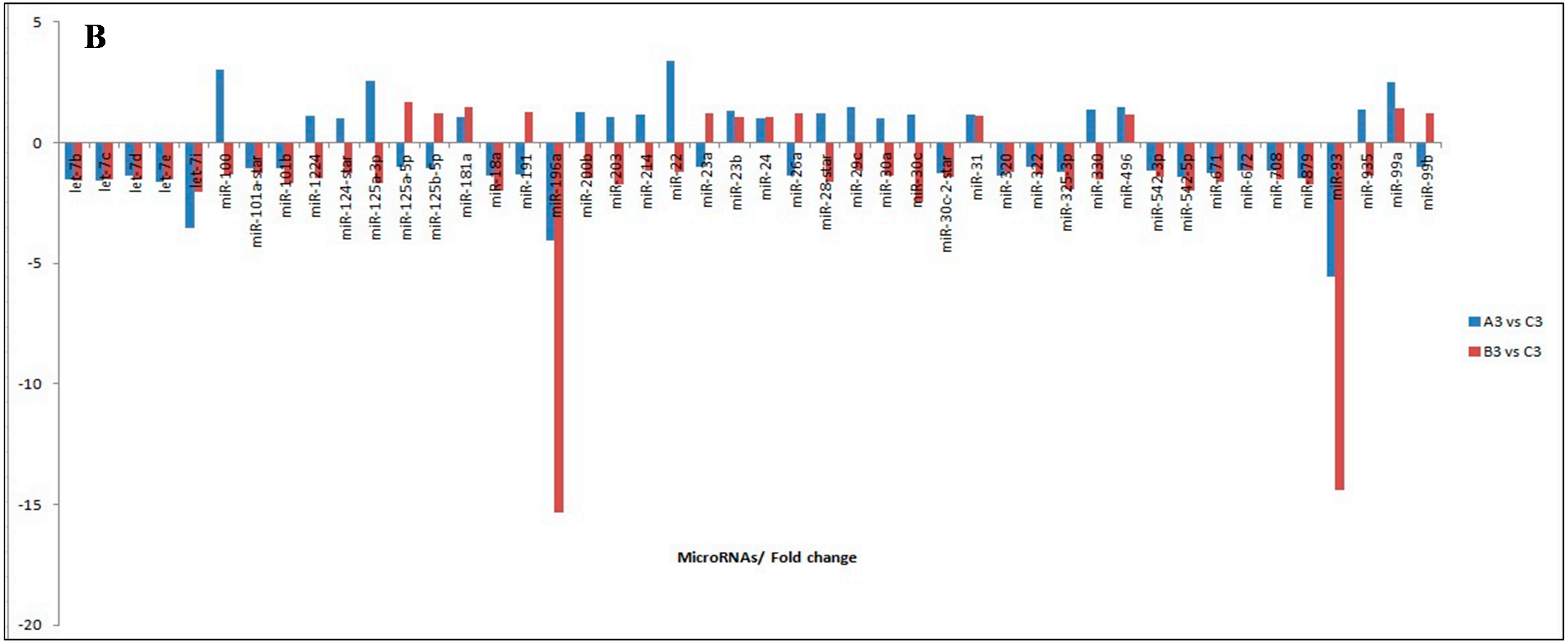

Figure S1. *Cont.*

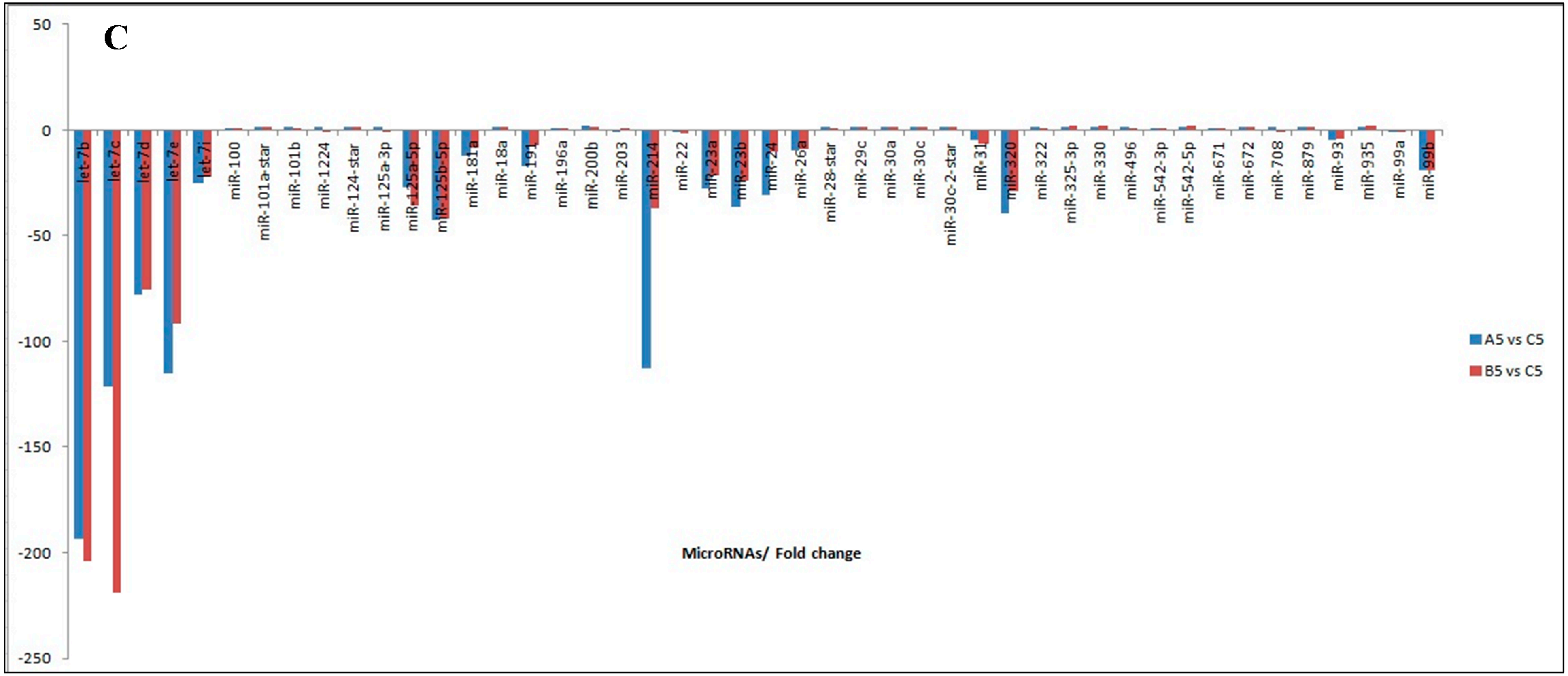

Figure S1. Significant expression changes in the microRNAs of Group A and Group B on the 1st day, 3rd day and 5th day after differentiation. (**A**) MicroRNAs expression on the 1st day; (**B**) MicroRNAs expression on the 3rd day; and (**C**) MicroRNAs expression on the 5th day.

Table S2. List of microRNAs with inconsistent expression throughout the experimental time intervals and excluded from the analysis.

Group A				Group B			
**miRNAs**	**Treatment Day**			**miRNAs**	**Treatment Day**		
	**1**	**3**	**5**		**1**	**3**	**5**
miR-101a*	1.96	−1.06	1.44	miR-100	4.53	1.14	1.06
miR-101b	2.38	−1.06	1.20	miR-101a*	3.18	−1.28	1.74
miR-181a	−3.81	1.08	−12.22	miR-101b	3.26	−1.68	1.11
miR-18a	2.76	−1.38	−1.27	miR-124*	4.36	−1.29	1.26
miR-196a	3.18	−4.08	1.11	miR-125a-5p	−3.92	1.67	−35.49
miR-214	−2.90	1.14	−113.07	miR-125b-5p	−1.53	1.20	−42.21
miR-23b	−2.87	1.31	−36.67	miR-181a	−2.07	1.46	−8.51
miR-24	−1.88	1.00	−31.09	miR-18a	4.79	−2.02	1.41
miR-30c-2*	2.10	−1.26	1.40	miR-191	−1.38	1.26	−7.24
miR-31	−2.45	1.16	−4.76	miR-200b	3.86	−1.46	1.76
miR-322	2.47	−1.03	1.15	miR-203	3.29	−1.72	1.03
miR-325-3p	2.44	−1.24	1.50	miR-23a	−5.61	1.23	−21.40
miR-542-3p	2.09	−1.15	1.06	miR-23b	−1.34	1.07	−24.30
miR-542-5p	1.14	−1.41	1.53	miR-26a	−1.80	1.24	−9.31
miR-671	1.40	−1.29	1.00	miR-28*	3.07	−1.64	1.02
miR-672	10.99	−1.18	1.74	miR-29c	5.44	−1.18	1.37
miR-708	1.74	−1.18	1.50	miR-30a	3.35	−1.39	1.26
miR-879	1.70	−1.49	1.66	miR-30c	2.48	−2.46	1.33
miR-22	1.14	3.40	−1.07	miR-30c-2*	1.71	−1.41	1.46
miR-99a	1.88	2.50	−1.04	miR-31	−134	1.12	−6.44
				miR-322	6.76	−1.34	1.11
				miR-325-3p	3.71	−1.92	1.77
				miR-330	4.10	−1.55	1.90
				miR-542-3p	5.28	−1.43	1.10
				miR-542-5p	3.38	−2.01	1.77
				miR-671	4.53	−1.63	1.08
				miR-672	4.07	−1.15	1.40
				miR-879	3.96	−1.72	1.63
				miR-935	5.90	−1.40	1.96
				miR-99a	−1.20	1.42	−1.13
				miR-99b	−4.57	1.21	−19.11

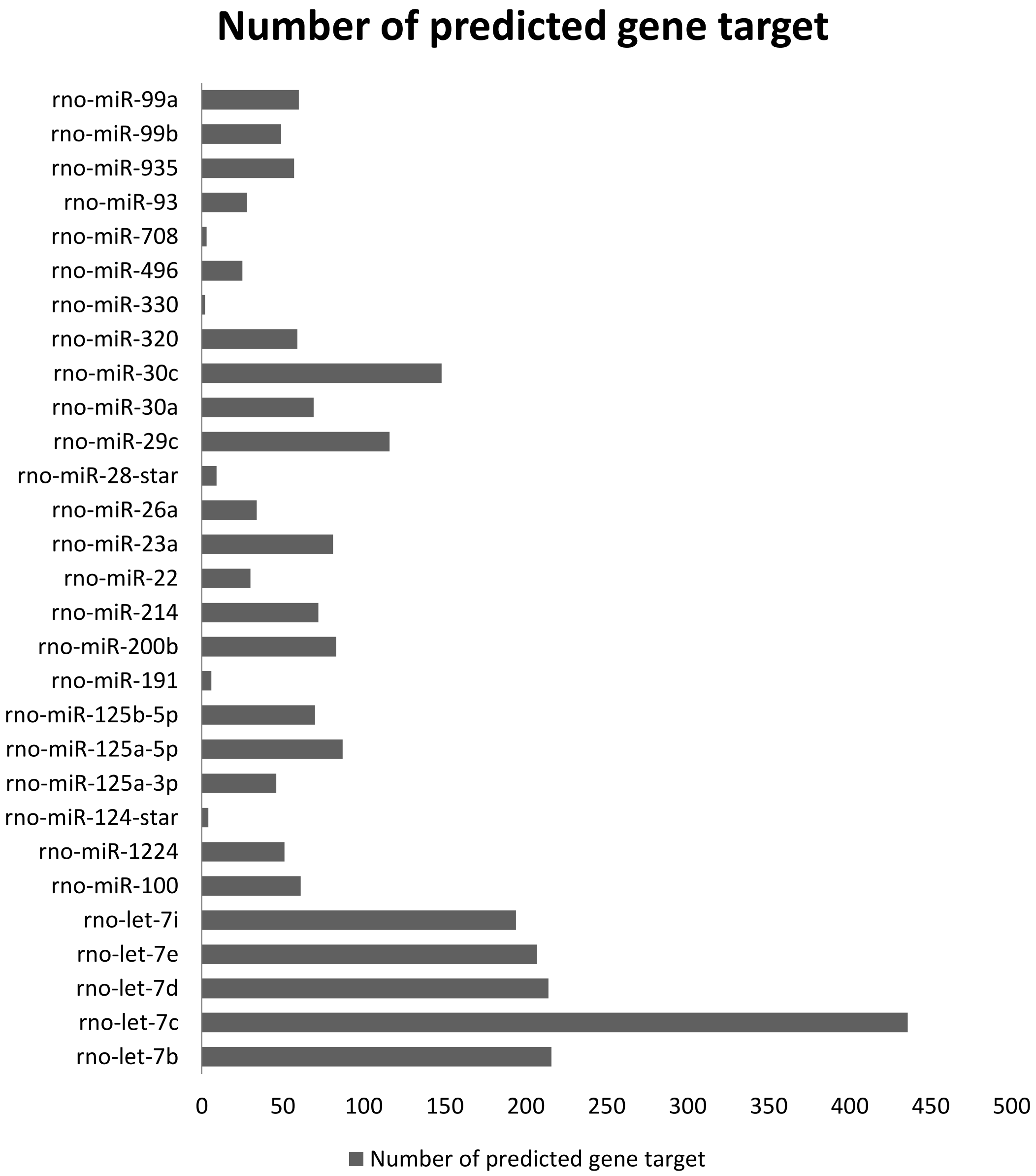

Figure S2. Number of predicted genes for each miRNA constantly up- and down-regulated in Group A (EGF + bFGF) and Group B (EGF + bFGF + IGF-1). Target genes were predicted by miRWalk database. The number of predicted gene target varied ranging from 2 (miR-330) to 436 (let-7c).

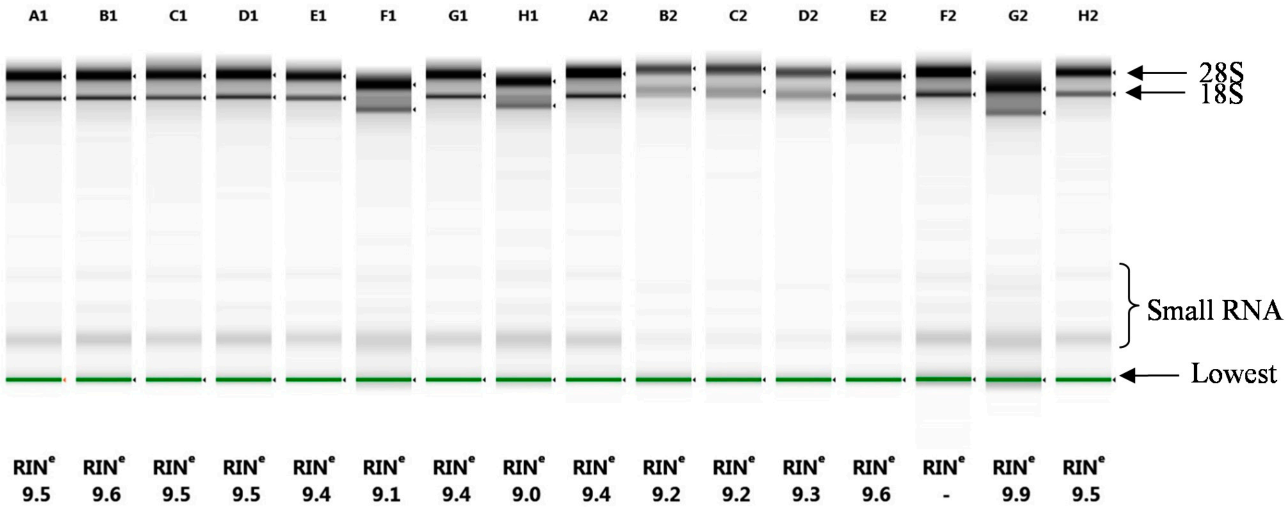

Figure S3. Gel snapshot of RNA samples by 2200 Agilent Tapestation System. Small RNA population including microRNAs were observed from the gel snapshot indicate that small saiz RNAs were well conserved during total RNA extraction.

Table S7. The quality of RNA and small RNA population. Quality of both RNA and detectable small RNA was determined by 2200 Agilent Tapestation System. Samples with RIN^e value more than 9.0 and 28S/18S ratio more than 1.2 were preceded for miRNA microarray screening.

Location ID	Sample	RIN^e Value	28S/18S Ratio
A1	Group A day 1	9.5	1.7
B1	Group A day 3	9.6	1.6
C1	Group A day 5 (rep 1)	9.5	1.8
D1	Group C day 1	9.5	1.7
E1	Group C day 3	9.4	2.0
F1	Group C day 5	9.1	2.9
G1	Group B day1 (rep 1)	9.4	1.6
H1	Group B day1 (rep 2)	9.0	2.7
A2	Group B day1 (rep 3)	9.4	1.8
B2	Group B day 3 (rep 1)	9.2	2.5
C2	Group B day 3 (rep 2)	9.2	2.5
D2	Group B day 3 (rep 3)	9.3	2.1
E2	Group B day 5 (rep 1)	9.6	2.8
F2	Group B day 5 (rep 2)	–	2.4
G2	Group B day 5 (rep 3)	9.9	2.8
H2	Group A day 5 (rep 2)	9.5	2.5
